# Comparison of subjective cognitive decline and polygenic risk score in the prediction of all-cause dementia, Alzheimer’s disease and vascular dementia

**DOI:** 10.1186/s13195-024-01559-9

**Published:** 2024-08-19

**Authors:** Kira Trares, Hannah Stocker, Joshua Stevenson-Hoare, Laura Perna, Bernd Holleczek, Konrad Beyreuther, Ben Schöttker, Hermann Brenner

**Affiliations:** 1 Division of Clinical Epidemiology and Aging Research, German Cancer Research Center, Im Neuenheimer Feld 581, 69120 Heidelberg, Germany; 2 Network Aging Research, Heidelberg University, Bergheimer Straße 20, 69115 Heidelberg, Germany; 3 Department Genes and Environment, Max Planck Institute of Psychiatry, Kraepelinstraße 2-10, 80804 Munich, Germany; 4 Saarland Cancer Registry, Neugeländstraße 9, 66117 Saarbrücken, Germany

**Keywords:** Subjective cognitive decline, Polygenic risk score, Risk prediction, Cohort study, Dementia, Alzheimer’s disease, Vascular dementia, CAIDE

## Abstract

**Background:**

Polygenic risk scores (PRS) and subjective cognitive decline (SCD) are associated with the risk of developing dementia. It remains to examine whether they can improve the established cardiovascular risk factors aging and dementia (CAIDE) model and how their predictive abilities compare.

**Methods:**

The CAIDE model was applied to a sub-sample of a large, population-based cohort study (*n* = 5,360; aged 50–75) and evaluated for the outcomes of all-cause dementia, Alzheimer’s disease (AD) and vascular dementia (VD) by calculating Akaike’s information criterion (AIC) and the area under the curve (AUC). The improvement of the CAIDE model by PRS and SCD was further examined using the net reclassification improvement (NRI) method and integrated discrimination improvement (IDI).

**Results:**

During 17 years of follow-up, 410 participants were diagnosed with dementia, including 139 AD and 152 VD diagnoses. Overall, the CAIDE model showed high discriminative ability for all outcomes, reaching AUCs of 0.785, 0.793, and 0.789 for all-cause dementia, AD, and VD, respectively. Adding information on SCD significantly increased NRI for all-cause dementia (4.4%, *p* = 0.04) and VD (7.7%, *p* = 0.01). In contrast, prediction models for AD further improved when PRS was added to the model (NRI, 8.4%, *p* = 0.03). When *APOE* ε4 carrier status was included (CAIDE Model 2), AUCs increased, but PRS and SCD did not further improve the prediction.

**Conclusions:**

Unlike PRS, information on SCD can be assessed more efficiently, and thus, the model including SCD can be more easily transferred to the clinical setting. Nevertheless, the two variables seem negligible if *APOE* ε4 carrier status is available.

**Supplementary Information:**

The online version contains supplementary material available at 10.1186/s13195-024-01559-9.

## Introduction

Fifty-five million people are living with dementia worldwide, and this number is projected to rise to nearly 80 million by 2030 [1]. However, according to the latest report of the Lancet standing Commission, up to 45% of all dementia cases can be prevented or delayed by altering 14 risk factors associated with daily living [2]. This indicates a high potential for early intervention and prevention.

To date, many different dementia risk prediction models have been developed based on varying requirements and settings. While some models are used as predictive models for long-term risk, others are used for diagnostic purposes or short-term risk prediction. Although such models can aid early detection of dementia, existing models require further improvement [3, 4]. For example, most existing models lack external validation [5–7]. Furthermore, since the field of dementia risk factors is highly dynamic and new factors keep being reported, dementia risk prediction models must be dynamic and easily modifiable [7, 8].

The Cardiovascular Risk Factor Aging and Dementia (CAIDE) model is an established and well-validated, multifactorial risk prediction model including modifiable as well as non-modifiable dementia risk factors [9]. In the same-titled development cohort, an AUC of 0.776 (95% confidence interval (CI): 0.717–0.836) was reached for predicting dementia diagnoses during 20 years of follow-up. Nevertheless, the CAIDE model only contains a general set of risk factors and might be further improved.

Previous studies have shown that patients reporting subjective cognitive decline (SCD), e.g., in the form of memory complaints, are at higher risk of mild cognitive impairment (MCI) and dementia [10–12]. It is assumed that SCD emerges 10–15 years before the onset of objective cognitive decline [12]. Thus, SCD is one of the earliest indicators of dementia [13]. In addition, the use of polygenic risk scores (PRSs) for risk prediction has been established during the last decades [14]. PRSs derived from genome-wide association studies (GWAS) calculate a person’s total genetic risk of disease and provide an emerging tool to differentiate the risk of developing AD at an individual level. They are thus commonly used for research purposes as well as in clinical settings [14–16]. Numerous studies have proven the successful prediction of the risk of developing dementia, specifically AD, by PRSs [16–19].

Therefore, this study aims to extend the well-established CAIDE model for dementia by PRS and SCD to assess as well as compare their predictive abilities for all-cause dementia, AD, and VD within 17 years of follow-up of a large population-based cohort of older adults. In addition, the models’ discrimination performances are evaluated in subgroups for mid-life (50–64) and late-life (65–75) to examine whether the performance of the extended models varies by age.

## Methods

### Study population

Analyses for this study were conducted using the ESTHER study. The ESTHER study (German name: Epidemiologische Studie zu Chancen der Verhütung, Früherkennung und optimierten Therapie chronischer Erkrankungen in der älteren Bevölkerung) is a prospective cohort study initiated between 2000 and 2002. Participants were recruited throughout Saarland, a German federal state, when visiting one of 420 cooperating physicians for a general health checkup at their general practitioners (GPs). The GPs asked their patients for consent to participate in the ESTHER study during this visit. Overall, the study comprises 9,940 participants aged 50 to 75, who were followed up 2, 5, 8, 11, 14, and 17 years after baseline. Details have been described elsewhere [20, 21]. Compared to a National Health Survey performed in a representative sample of the German population in 1998, sociodemographic baseline characteristics were similarly distributed in the respective age categories of the ESTHER study [22]. The ethics committees of the Heidelberg Medical Faculty of Heidelberg University and the state medical board of Saarland, Germany, approved the study.

### Dementia assessment and study sample

Dementia diagnoses were assessed during the 14- and 17-year follow-ups of the ESTHER study. The mean follow-up time was 14.8 years (± SD 3.5 years) with a maxiumum of 19.8 years due to the duration of 2 years of baseline recruitment. Standardised questionnaires were sent to the study participants’ GPs, including whether they were aware of a dementia diagnosis of their patient. If so, the GPs were asked to provide details such as the type of dementia, the date of diagnosis, and all available medical records from other specialised providers like neurologists or memory clinics. Questionnaires were also sent to the GPs of participants lost to follow-up due to ill health or death.

**Fig. 1:**
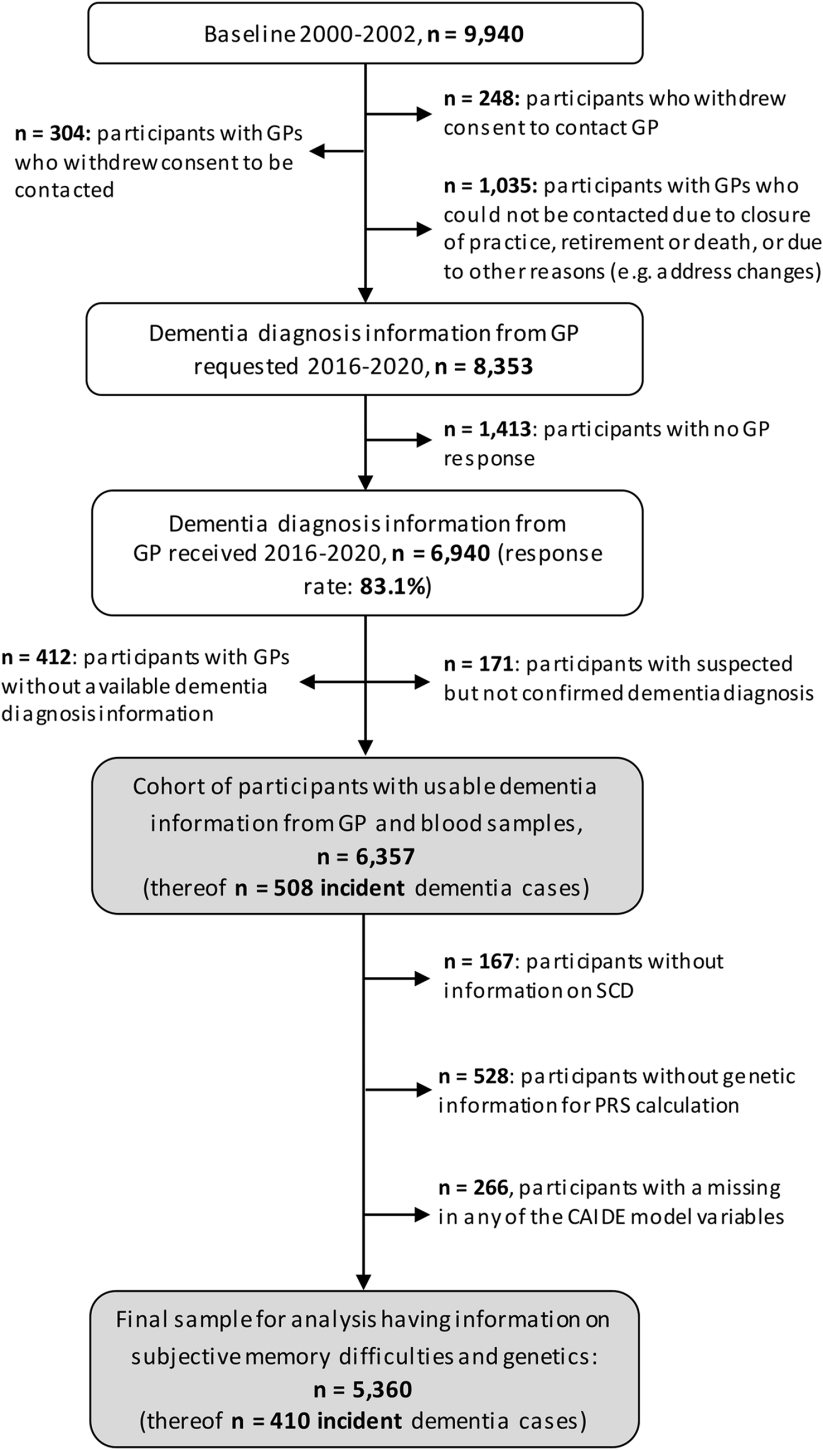
Flowchart of selected study participants based on the ESTHER study. Abbreviations: GP, general practitioner; SCD, subjective cognitive decline; PRS, polygenic risk score, CAIDE, Cardiovascular Risk Factors Aging and Dementia

Information about a dementia diagnosis was received from 6,357 ESTHER study participants (Fig. 1). For the outlined analyses, participants without information on SCD (*n* = 167), without genetic information for PRS calculation (*n* = 528), and with missing values in any of the CAIDE model variables (*n* = 266) were excluded. Hence, the study sample comprised 5,360 study participants.

### CAIDE model and covariates

The CAIDE model is an established and well-validated dementia risk prediction model developed using a sample of 1,409 participants of the CAIDE study [9]. The CAIDE study is a Finnish population-based cohort study investigating cardiovascular risk factors, aging and dementia. Participants aged 39 to 64 at baseline were followed up for 20 years. During this follow-up period, 61 participants developed dementia. The CAIDE model includes age, education, sex, systolic blood pressure, body mass index (BMI), total cholesterol, and physical activity. A second version of the model (CAIDE Model 2) also comprises participants’ *APOE* ε4 carrier status.

All variables included in the CAIDE model were available in the ESTHER study. Categorization of variables had to be recalibrated due to differences in the age range, the education system and the assessment of physical activity between the studies. In detail, age was used as a continuous variable, education categories have been changed from 0 to 6, 7–9, ≥ 10 years to ≤ 9, 10–11, ≥ 12 years, and physical activity has been changed from two categories (inactive and active) to three categories (inactive, low, and medium or high). Furthermore, the continuous variables age, systolic blood pressure, BMI, and total cholesterol were tested for their best-fitting function using fractional polynomials [23]. Since the linear function was the best-fitting function for all variables in predicting all-cause dementia, AD, and VD (data not shown), they were kept as continuous variables.

Information about age, sex, education, BMI, and physical activity was collected via standardised self-administered questionnaires during the baseline assessment of the ESTHER study, while the systolic blood pressure was measured by the participants’ GPs. Serum samples drawn at baseline were used to measure the study participant’s total cholesterol levels by an enzymatic colorimetric test with the Synchron LX multicalibrator system (Beckman Coulter, Galway, Ireland). To determine *APOE* genotypes, DNA was extracted from whole blood samples through a sorting-out procedure. Genotyping of blood cell-derived DNA was carried out using the Illumina Infinium OncoArray and Global Screening Array BeadChips (Illumina, San Diego, CA, USA). Quality control assessment was performed according to the Nature Protocols article by Anderson and colleagues [24]. The Michigan Imputation Server was utilized for the imputation of the quality-controlled data. For this, SHAPEIT2 was used to phase the data, and MiniMac 4 was used to impute to the HRC Version r1.1 2016 reference panel [25, 26]. *APOE* genotypes were finally determined using TaqMan single-nucleotide polymorphism (SNP) assays (Applied Biosystems, California, USA) were used. Missing APOE information (5% of *APOE* data) was imputed based on quality-controlled imputed genetic data. Further details have been described elsewhere [27].

### Polygenic risk score calculation and subjective cognitive decline

PRS were calculated based on AD-associated SNPs identified by Kunkle et al., with a GWAS significance threshold of *p* < 5*10^− 8^ [28]. For this purpose, the number of risk alleles was summed and weighted according to the extent of the association as previously described in detail [17]. SNPs in the APOE locus (chromosome 19, 45,404,000–45,418,000) were not included in this PRS.

SCD was assessed via self-administered health questionnaires during the baseline assessment, including a yes or no question regarding short-term memory complaints (*Do you have difficulty remembering things that have happened in the recent past (hours to a few days)?*).

### Statistical analyses

Baseline characteristics of included ESTHER study participants were calculated for participants with incident all-cause dementia, AD, and VD diagnosis as well as participants without dementia diagnosis during follow-up. Additional comparisons of, baseline characteristics of ESTHER study participants included and excluded from the analyses showed reasonably comparable results (Supplemental Table 1).

Cox proportional hazard regression models adjusted for age, sex, education, systolic blood pressure, BMI, total cholesterol, physical activity and *APOE* ε4 carrier status were calculated to assess the association between CAIDE model variables and the outcomes of all-cause dementia, AD, and VD. Furthermore, the associations between the predictors of interest (PRS and SCD) and the three dementia outcomes were examined. Statistical significance was assessed by the Wald test.

The discriminative ability of CAIDE Model 1 and CAIDE Model 2 for all-cause dementia, AD, and VD was determined by Akaike’s information criterion (AIC) and the area under the receiver operating characteristic (ROC) curve (AUC) and 95% confidence intervals (CIs). For this purpose, the “survcstd” macro of SAS was used. Model calibration of all prediction models was examined by May-Hosmer’s simplification of the Gronnesby-Borgan test [29]. To examine the improvement of the CAIDE Model by PRS and SCD, the net reclassification improvement (NRI) method was applied [30, 31]. For this, three cut-offs were chosen individually for each outcome with an equal distribution of incident dementia diagnoses and applied to the analyses. In addition, the extent of the model’s improvement was assessed by the integrated discrimination improvement (IDI) [30, 31]. In both methods, CAIDE Model 1 and CAIDE Model 2 were used as reference models, and PRS and SCD were added to the models. The latter was modelled as a binary categorical variable, while the PRS was modelled continuously (per one standard deviation increase). Analyses were carried out in the total sample, as well as in subgroups for mid-life (50–64 years) and late-life (65–75 years) for all outcomes and CAIDE Model 1 and 2, respectively.

All analyses were conducted using the Statistical Analysis System (SAS, version 9.4, Cary, North Carolina, USA). Statistical tests were two-sided using an alpha level of 0.05.

## Results

Baseline characteristics for all variables included in the basic CAIDE model are described separately for participants with incident all-cause dementia, AD, and VD diagnosis as well as for those without a dementia diagnosis in Table 1. Among the included 5,360 study participants, 410 were diagnosed with dementia. Of those, 139 participants had an AD diagnosis, and 152 were diagnosed with VD. The mean age at baseline of participants who later developed incident all-cause dementia was five years higher (mean (± SD): 66.3 (5.2) years) compared to participants without dementia diagnosis (61.7 (6.5) years). Most participants (> 70%) had an education of no more than 9 years. Systolic blood pressure, BMI and total cholesterol levels of study participants were comparable between participants with and without later dementia diagnoses. Those who were later diagnosed with dementia more frequently reported low or inactive levels of physical activity than those with no dementia diagnosis (71.2% compared to 62.9%). Finally, the proportion of *APOE* ε4 carriers was much higher among those who later developed dementia, in particular among those who were diagnosed with AD (50.4%), than among participants with no dementia diagnosis (25.0%).

**Table 1:** CAIDE model variables of the study population (*n* = 5,360)

CAIDE model variables	No dementia (*n* = 4,950)		All-cause dementia (*n* = 410)			Alzheimer’s disease (*n* = 139)			Vascular dementia (*n* = 152)	
	Data		Data	*p*-value^a^		Data	*p*-value^a^		Data	*p*-value^a^
**Age (years)**,** mean (SD)**	61.2 (6.4)		66.9 (5.2)	**< 0.001**		66.7 (5.2)	**< 0.001**		66.9 (5.0)	**< 0.001**
Mid-life (50–64 years), n (%)	3330 (67.3)		128 (31.2)			46 (33.1)			47 (30.9)	
Late-life (65–75 years), n (%)	1620 (32.7)		282 (68.8)			93 (66.9)			105 (69.1)	
**Education (years)**,** n (%)**				**0.001**			**0.036**			0.135
≤9	3592 (72.6)		331 (80.7)			115 (82.7)			122 (80.3)	
10–11	752 (15.2)		38 (9.3)			13 (9.4)			16 (10.5)	
≥ 12	606 (12.2)		41 (10.0)			11 (7.9)			14 (9.2)	
**Sex**,** n (%)**				0.072			0.968			0.076
Female	2680 (54.1)		203 (49.5)			75 (54.0)			71 (46.7)	
Male	2270 (45.9)		207 (50.5)			64 (46.0)			81 (53.3)	
**SBP (mmHg)**,** mean (SD)**	138.9 (19.5)		142.4 (19.2)	**< 0.001**		142.2 (19.2)	**0.046**		142.8 (19.5)	**0.007**
**BMI (kg/m**^**2**^**)**,** mean (SD)**	27.7 (4.4)		27.6 (3.9)	0.924		27.1 (3.8)	0.304		27.7 (4.0)	0.957
**Total cholesterol (mmol/L)**, **mean (SD)**	5.69 (1.3)		5.69 (1.3)	0.975		5.73 (1.3)	0.545		5.68 (1.3)	0.925
**Physical activity**^**b**^, **n (%)**				**< 0.001**			**0.001**			0.178
Inactive	830 (16.8)		108 (26.3)			43 (30.9)			34 (22.4)	
Low	2284 (46.1)		184 (44.9)			57 (41.0)			71 (46.7)	
Medium or high	1836 (37.1)		118 (28.8)			39 (28.1)			47 (30.9)	
***APOE *genotypes**,** n (%)**				**< 0.001**			**< 0.001**			**0.014**
ε4 non-carrier	3710 (75.0)		243 (59.3)			69 (49.6)			99 (65.1)	
ε4 carrier	1240 (25.0)		167 (40.7)			70 (50.4)			53 (34.9)	

Abbreviations: *APOE*, apolipoprotein E; SBP, systolic blood pressure; BMI, body mass index

Note: Numbers printed in bold are statistically significant.

^a^ Result of statistical test (Mann-Whitney-U, Wilcoxon Rank-sum, or χ^2^ test as appropriate) for comparison with group “No dementia”.

^b^ “Inactive” was defined by < 1 h of vigorous or < 1 h light physical activity per week. “Medium or high” was defined by ≥ 2 h of vigorous and ≥ 2 h of light physical activity/week. All other amounts of physical activity were grouped into the category “Low”.

In a Cox regression model adjusted for all CAIDE model variables, age and *APOE* ε4 carrier status in Model 2 were statistically significantly associated with all dementia outcomes (Supplemental Tables 2–4). In addition, higher education (inverse), male sex, and higher physical activity (inverse) showed significant associations with all-cause dementia (Supplemental Table 2). In analyses focusing on the AD outcome, only physical activity was significantly associated (Supplemental Table 3). In analyses focusing on VD, only male sex was significantly associated (Supplemental Table 4).

Cox regression models further showed significant associations of PRS and SCD with all-cause dementia in Model 1 (Table 2). When *APOE* ε4 carrier status was added in Model 2, the association between PRS and all-cause dementia lost statistical significance, but SCD remained a significant predictor. For dementia subtypes, PRS was statistically significantly associated with AD, while SCD was statistically significantly associated with VD.

**Table 2:** Associations between predictors of interest and common subtypes of dementia

Predictors	*n*_total_	*n*_cases_	Model 1^a^			Model 2^b^	
			HR (95% CI)	*p*-value		HR (95% CI)	*p*-value
**All-cause dementia**							
SCD	5,360	410	**1.49 (1.21–1.82)**	**< 0.001**		**1.48 (1.21–1.82)**	**< 0.001**
Kunkle PRS per 1SD^c^			**1.22 (1.11–1.34)**	**< 0.001**		1.03 (0.93–1.15)	0.350
**Alzheimer’s disease**							
SCD	5,089	139	1.27 (0.89–1.83)	0.191		1.27 (0.88–1.83)	0.197
Kunkle PRS per 1SD^d^			**1.51 (1.29–1.76)**	**< 0.001**		**1.20 (1.00-1.43)**	**0.049**
**Vascular dementia**							
SCD	5,102	152	**1.62 (1.16–2.25)**	**0.005**		**1.62 (1.16–2.25)**	**0.005**
Kunkle PRS per 1SD^e^			1.04 (0.88–1.23)	0.680		0.91 (0.76–1.09)	0.291

Note: Numbers printed in bold are statistically significant (*P* < 0.05)

Abbreviations: HR, hazard ratio; CI, confidence interval; SD, standard deviation; SCD, subjective cognitive decline; PRS, polygenic risk score;

^a^Model 1 was adjusted for age, education, sex, systolic blood pressure, BMI, total cholesterol and physical activity

^b^Model 2 was adjusted for all variables listed in Model 1 and *APOE* ε4 carrier status

^c^1 SD of Kunkle’s PRS = 0.0100

^d^1 SD of Kunkle’s PRS = 0.0010

^e^1 SD of Kunkle’s PRS = 0.0099

The performance of CAIDE Models 1 and 2 for all-cause dementia, AD, and VD are shown in Table 3. May-Hosmer’s simplification of the Gronnesby-Borgan test verified good calibration for all models (data not shown). Overall, the CAIDE model showed a high discriminative ability in the total population with an AUC > 0.78 in Model 1. AUCs further improved to 0.800, 0.827, and 0.793 for the outcomes of all-cause dementia, AD, and VD, respectively, when *APOE* ε4 carrier status was added to the CAIDE model (Model 2).

**Table 3:** Evaluation of prediction models for all-cause dementia, Alzheimer’s disease and vascular dementia

Model performance measures	Model 1^a^				Model 2^b^		
	CAIDE	CAIDE + PRS	CAIDE + SCD		CAIDE	CAIDE + PRS	CAIDE + SCD
**All-cause dementia (** ***n*** ** = 410)**							
**AIC**	6364.8	6351.6	6352.9		6318.2	6319.9	6306.5
**AUC (95% CI)**	0.785 (0.765–0.806)	0.790 (0.769–0.810)	0.788 (0.768–0.809)		0.800 (0.780–0.820)	0.800 (0.780–0.820)	0.803 (0.782–0.823)
**Reclassification**							
Events n_up_/n_down_	Ref.	37/29	44/30		Ref.	6/4	40/29
Nonevents n_up_/n_down_	Ref.	237/264	258/307		Ref.	37/45	244/266
NRI % ^c^ (*p*-value)	Ref.	2.5% (0.219)	**4.4% (0.040)**		Ref.	0.6% (0.335)	3.1% (0.064)
IDI % (*p*-value)	Ref.	0.07% (0.070)	**0.04% (0.011)**		Ref.	0.005% (0.968)	**0.05% (0.008)**
**Alzheimer’s disease (** *n* ** = 139)**							
**AIC**	2163.7	2141.0	2164.1		2124.0	2122.2	2124.4
**AUC (95% CI)**	0.793 (0.758–0.827)	0.808 (0.774–0.841)	0.793 (0.759–0.827)		0.827 (0.795–0.859)	0.827 (0.795–0.859)	0.827 (0.795–0.859)
**Reclassification**							
Events n_up_/n_down_	Ref.	28/20	7/7		Ref.	8/9	7/3
Nonevents n_up_/n_down_	Ref.	396/525	143/162		Ref.	181/193	138/153
NRI % ^d^ (*p*-value)	Ref.	**8.4% (0.026)**	0.4% (0.859)		Ref.	-0.5% (0.841)	3.2% (0.128)
IDI % (*p*-value)	Ref.	**0.05% (0.030)**	-0.004% (0.510)		Ref.	0.01% (0.132)	0.0003% (0.296)
**Vascular dementia (** *n* ** = 152)**							
**AIC**	2331.9	2363.7	2356.3		2355.4	2356.3	2349.8
**AUC (95% CI)**	0.789 (0.757–0.822)	0.790 (0.757–0.822)	0.794 (0.761–0.828)		0.793 (0.760–0.826)	0.794 (0.761–0.827)	0.798 (0.764–0.831)
**Reclassification**							
Events n_up_/n_down_	Ref.	4/2	24/14		Ref.	7/7	17/18
Nonevents n_up_/n_down_	Ref.	45/32	287/344		Ref.	99/97	289/317
NRI % ^e^ (*p*-value)	Ref.	1.1% (0.316)	**7.7% (0.010)**		Ref.	-0.04% (0.981)	-0.09% (0.975)
IDI % (*p*-value)	Ref.	0.002% (0.837)	0.003% (0.093)		Ref.	-0.004% (0.359)	-0.007% (0.068)

Note: Numbers printed in bold are statistically significant (*P* < 0.05)

Abbreviations: SCD, subjective cognitive decline; PRS, polygenic risk score; AIC, Akaike’s information criterion; AUC, area under the curve; NRI, net reclassification improvement; IDI, integrated discrimination improvement

^a^CAIDE Model 1 includes age, education, sex, systolic blood pressure, BMI, total cholesterol and physical activity

^b^CAIDE Model 2 includes all variables listed in Model 1 and *APOE* ε4 carrier status

^c^The cutoffs for all-cause dementia were set to 7%, 15%, and 30%

^d^The cutoffs for AD were set to 3%, 6%, and 11%

^e^The cutoffs for VD were set to 3.5%, 7%, and 14%

Generally, the extension of CAIDE Model 1 by PRS and SCD only led to marginal improvements in AUCs. However, IDI and NRI revealed statistically significant improvements for some models. Adding SCD to CAIDE Model 1 led to a statistically significant improvement of NRI (4.4%, *p* = 0.04) and IDI in predicting all-cause dementia. In contrast, in the case of AD, extending CAIDE Model 1 by PRS but not SCD revealed statistically significantly better prediction with an NRI of 8.4% (*p* = 0.03) and significant IDI. For VD, the extension by SCD again led to a significant improvement of the NRI (7.7%, *p* = 0.01) but IDI was not statistically significant.

When *APOE* ε4 carrier status was included in CAIDE Model 2, AUCs did not further improve by adding PRS and SCD to the models. Also, NRI and IDI statistics showed no statistically significant changes except for CAIDE Model 2 plus SCD for the outcome of all-cause dementia. In this case, IDI was statistically significant, and NRI showed an improvement of 3.1% but did not reach statistical significance (*p* = 0.06).

In addition to the total population, the discriminative abilities of the CAIDE model were evaluated in mid-life (50–64 years) and late-life (65–75 years) subgroups (Tables 4, 5 and 6). Overall, AUCs were consistently higher in the mid-life compared to the late-life subgroup for all outcomes. In contrast to the total population, PRS significantly improved the prediction of CAIDE Model 1 for all-cause dementia in mid-life with an NRI of 6.9% (*p* = 0.008) (Table 4). In addition, as observed in the total population, the extension by SCD also led to a significant improvement in IDI for all-cause dementia in this subgroup. However, the increase in NRI was only modest in this case and not statistically significant (2.0%, *p* = 0.62). The overall strongest improvement of the CAIDE model was achieved for AD when CAIDE model 1 was extended by PRS in the mid-life subgroup (NRI, 19.6%, *p* = 0.008) (Table 5). Also, IDI was statistically significant. For the outcome of VD, SCD led to significant improvements in NRI but not IDI in the late-life subgroup for CAIDE Model 1, whereas PRS led to a statistically significant NRI but not IDI in CAIDE Model 2 in both agegroups (Table 6).

**Table 4:** Evaluation of the discriminative ability for all-cause dementia in mid-life and late-life

Model performance measures	Model 1^a^				Model 2^b^		
	CAIDE	CAIDE + PRS	CAIDE + SCD		CAIDE	CAIDE + PRS	CAIDE + SCD
^**a**^**Mid-life (50–64 years)***[n*_*total*_*=3458*,* n*_*cases*_*=128]*							
**AIC**	1930.1	1919.4	1925.5		1908.0	1908.7	1903.3
**AUC (95% CI)**	0.731 (0.692–0.771)	0.744 (0.704–0.784)	0.735 (0.696–0.774)		0.758 (0.718–0.797)	0.759 (0.719–0.799)	0.760 (0.720–0.800)
**Reclassification**							
Events n_up_/n_down_	Ref.	25/21	23/24		Ref.	8/6	20/20
Nonevents n_up_/n_down_	Ref.	266/392	283/377		Ref.	106/112	260/321
NRI %^c^ (*p*-value)	Ref.	**6.9% (0.008)**	2.0% (0.616)		Ref.	1.7% (0.455)	1.8% (0.631)
IDI % (*p*-value)	Ref.	**0.03% (0.018)**	**0.02% (0.018)**		Ref.	-0.0007% (0.798)	**0.02% (0.021)**
^**b**^**Late-life (65–75 years)***[n*_*total*_*=1902*,* n*_*cases*_*=282]*							
**AIC**	3958.0	3955.4	3952.7		3935.5	3937.4	3930.4
**AUC (95% CI)**	0.673 (0.641–0.705)	0.678 (0.646–0.710)	0.678 (0.647–0.710)		0.697 (0.666–0.728)	0.697 (0.666–0.728)	0.699 (0.669–0.730)
**Reclassification**							
Events n_up_/n_down_	Ref.	23/23	39/41		Ref.	1/2	24/35
Nonevents n_up_/n_down_	Ref.	98/127	180/182		Ref.	3/4	156/164
NRI %^d^ (*p*-value)	Ref.	1.8% (0.462)	-0.59% (0.851)		Ref.	-0.29% (0.531)	-3.4% (0.237)
IDI % (*p*-value)	Ref.	0.12% (0.119)	**0.05% (0.031)**		Ref.	-0.003% (0.874)	**0.07% (0.023)**

Note: Numbers printed in bold are statistically significant (*P* < 0.05)

Abbreviations: SCD, subjective cognitive decline; PRS, polygenic risk score; AIC, Akaike’s information criterion; AUC, area under the curve; NRI, net reclassification improvement; IDI, integrated discrimination improvement

^a^CAIDE Model 1 includes age, education, sex, systolic blood pressure, BMI, total cholesterol and physical activity

^b^CAIDE Model 2 includes all variables listed in Model 1 and *APOE* ε4 carrier status

^c^The cutoffs for mid-life were set to 4.25%, 6.5%, and 10%

^d^The cutoffs for late-life were set to 15%, 22.5%, and 35%

**Table 5:** Evaluation of the discriminative ability for Alzheimer’s disease in mid-life and late-life

Model performance measures	Model 1^a^				Model 2^b^		
	CAIDE	CAIDE + PRS	CAIDE + SCD		CAIDE	CAIDE + PRS	CAIDE + SCD
^**a**^**Mid-life (50–64 years)***[n*_*total*_*=3330*,* n*_*cases*_*=46]*							
**AIC**	698.6	680.4	699.2		677.5	674.5	677.9
**AUC (95% CI)**	0.767 (0.705–0.829)	0.794 (0.734–0.854)	0.765 (0.703–0.827)		0.809 (0.743–0.875)	0.811 (0.747–0.875)	0.808 (0.743–0.873)
**Reclassification**							
Events n_up_/n_down_	Ref.	12/6	5/2		Ref.	7/5	3/6
Nonevents n_up_/n_down_	Ref.	299/516	160/156		Ref.	175/230	113/119
NRI %^c^ (*p*-value)	Ref.	**19.6% (0.008)**	6.4% (0.163)		Ref.	6.0% (0.250)	-6.3% (0.110)
IDI % (*p*-value)	Ref.	**0.02% (0.025)**	-0.0006 (0.243)		Ref.	0.002% (0.239)	-0.001 (0.504)
^**b**^**Late-life (65–75 years)***[n*_*total*_*=1713*,* n*_*cases*_*=93]*							
**AIC**	1306.4	1301.5	1307.7		1290.1	1291.7	1291.5
**AUC (95% CI)**	0.689 (0.633–0.745)	0.708 (0.652–0.764)	0.688 (0.633–0.744)		0.740 (0.688–0.791)	0.738 (0.686–0.791)	0.739 (0.687–0.790)
**Reclassification**							
Events n_up_/n_down_	Ref.	16/15	4/9		Ref.	3/4	1/7
Nonevents n_up_/n_down_	Ref.	189/218	84/72		Ref.	40/51	65/88
NRI %^d^ (*p*-value)	Ref.	2.9% (0.595)	-6.1% (0.068)		Ref.	-4.0% (0.877)	-5.0% (0.101)
IDI % (*p*-value)	Ref.	0.06% (0.109)	-0.01% (0.683)		Ref.	0.01% (0.199)	-0.004% (0.390)

Note: Numbers printed in bold are statistically significant (*P* < 0.05)

Abbreviations: SCD, subjective cognitive decline; PRS, polygenic risk score; AIC, Akaike’s information criterion; AUC, area under the curve; NRI, net reclassification improvement; IDI, integrated discrimination improvement

^a^CAIDE Model 1 includes age, education, sex, systolic blood pressure, BMI, total cholesterol and physical activity

^b^CAIDE Model 2 includes all variables listed in Model 1 and *APOE* ε4 carrier status

^c^The cutoffs for mid-life were set to 1.5%, 3.5%, and 7%

^d^The cutoffs for late-life were set to 5%, 8%, and 15%

**Table 6:** Evaluation of the discriminative ability for vascular dementia in mid-life and late-life

Model performance measures	Model 1^a^				Model 2^b^		
	CAIDE	CAIDE + PRS	CAIDE + SCD		CAIDE	CAIDE + PRS	CAIDE + SCD
**Mid-life (50–64 years)***[n*_*total*_*=3377*,* n*_*cases*_*=47]*							
**AIC**	707.6	709.3	709.4		708.9	710.9	710.8
**AUC (95% CI)**	0.757 (0.703–0.812)	0.759 (0.702–0.815)	0.758 (0.703–0.812)		0.762 (0.706–0.817)	0.761 (0.705–0.818)	0.762 (0.706–0.818)
**Reclassification**							
Events n_up_/n_down_	Ref.	4/1	3/4		Ref.	4/1	5/3
Nonevents n_up_/n_down_	Ref.	99/99	115/110		Ref.	26/39	93/122
NRI %^c^ (*p*-value)	Ref.	6.38% (0.076)	-2.3% (0.554)		Ref.	**6.8% (0.001)**	5.1% (0.174)
IDI % (*p*-value)	Ref.	0.00007 (0.357)	-0.0005 (0.562)		Ref.	-0.0003 (0.451)	-0.0006 (0.588)
**Late-life (65–75 years)***[n*_*total*_*=1725*,* n*_*cases*_*=105]*							
**AIC**	1475.2	1477.2	1468.5		1469.4	1469.7	1462.9
**AUC (95% CI)**	0.669 (0.616–0.721)	0.669 (0.616–0.721)	0.694 (0.643–0.745)		0.683 (0.628–0.737)	0.683 (0.628–0.738)	0.702 (0.649–0.755)
**Reclassification**							
Events n_up_/n_down_	Ref.	1/1	29/19		Ref.	12/3	25/17
Nonevents n_up_/n_down_	Ref.	9/3	283/334		Ref.	98/107	288/317
NRI %^d^ (*p*-value)	Ref.	-0.4% (0.683)	**12.7% (0.043)**		Ref.	**9.1% (0.011)**	9.4% (0.127)
IDI % (*p*-value)	Ref.	0.002% (0.911)	0.01% (0.134)		Ref.	-0.01% (0.444)	0.3% (0.088)

Note: Numbers printed in bold are statistically significant (*P* < 0.05)

Abbreviations: SCD, subjective cognitive decline; PRS, polygenic risk score; AIC, Akaike’s information criterion; AUC, area under the curve; NRI, net reclassification improvement; IDI, integrated discrimination improvement

^a^CAIDE Model 1 includes age, education, sex, systolic blood pressure, BMI, total cholesterol and physical activity

^b^CAIDE Model 2 includes all variables listed in Model 1 and *APOE* ε4 carrier status

^c^The cutoffs for mid-life were set to 1.75%, 2.5%, and 3.5

^d^The cutoffs for late-life were set to 6.25%, 10%, and 15.5%

## Discussion

In this prospective cohort study, we evaluated potential improvement in dementia prediction with the established and well-validated CAIDE model by adding information about a PRS and SCD. While the PRS significantly improved the prediction of AD only, information on SCD significantly improved the predictive ability of the CAIDE Model 1 for all-cause dementia and VD. However, no relevant improvement in prediction was achieved when *APOE* ε4 carrier status was included in CAIDE Model 2.

### Previous studies

Very few dementia risk prediction models include information on SCD. The self-administered Gerocognitive Examination (SAGE) score is a cognitive assessment tool for mild cognitive impairment (MCI) and early dementia, including information on subjective cognitive decline (“Have you had any problems with memory or thinking?” Yes / only occasionally / no) as well as measures of cognitive function of different domains [32]. In its development cohort of 254 participants (> 59 years) from the cohort as well as the clinical setting, the model predicted the risk of developing dementia well with an AUC of 0.906. In a recent study, the authors tested the SAGE score compared to the Mini-Mental State Examination (MMSE) in 424 individuals and showed that SAGE predicts cognitive decline at least 6 months earlier than MMSE [33].

In the study of Licher and colleagues, a dementia risk prediction model was developed in a cohort based on the Rotterdam Study, including 20,324 individuals aged 60 and older [34]. The model includes subjective memory decline, age, history of stroke, and need for assistance with finances or medication. It predicted the risk of developing dementia with an AUC of 0.78 (95% CI: 0.75–0.81) and was externally validated in the Epidemiological Prevention Study of Zoetermeer (EPOZ), achieving a comparable predictive ability with an AUC of 0.75 (95% CI: 0.67–0.82).

PRSs are a widely used tool to assess an individual’s risk of developing dementia, especially AD [14, 15]. Nevertheless, multifactorial dementia risk prediction models, including such a score, are still rare. In a study by Escott-Price and colleagues, several models, including information on polygenic scores, were tested in a subset of 4.603 participants from the International Genomics of Alzheimer’s Project (IGAP) [18]. The model including the variables *APOE*, PRS, sex, and age, predicted the risk of developing AD the best, reaching an AUC of 0.78 (95% CI: 0.77–0.80).

Furthermore, Verhaaren et al. added a set of 10 risk genes to a model composed of age, sex, and *APOE* ε4 [35]. Like in our study, the AUC increased only marginally from 0.815 to 0.816. However, the authors did not apply further reclassification methods like NRI or IDI to determine the extent of improvement by the added risk genes.

### Interpretation of findings

The reclassification analyses showed that SCD significantly improved the prediction of the CAIDE model for all-cause dementia and VD. This result was further supported by Cox regression analyses showing a statistically significant association between SCD and all-cause dementia as well as VD. In the literature, participants reporting SCD have been shown to be at a higher risk of developing MCI and following dementia in a multitude of studies [10, 36, 37]. Meta-analyses revealed an annual conversion rate to MCI of 7% and to dementia of 5% [36]. Although SCD can be assessed in different ways, this does not appear to affect its effectiveness in predcting dementia risk [11, 37]. In our study, SCD was assessed by asking only one simple question to the participant. This is a major advantage in clinical translation of the created prediction model. In a clinical setting, obtaining all the necessary information to estimate an individual’s risk of developing dementia with the created prediction model would only require a concise physical examination and a patient interview, along with the submission of a blood sample. In addition, a further advantage of adding SCD to the CAIDE model is that SCD is one of the earliest indicators that appears even before cognitive decline can be objectively measured [11, 38]. This makes early intervention and prevention possible.

In contrast to SCD, PRS calculation needs more resources. We found that adding PRS to the CAIDE model significantly improved prediction for AD only. This was even more distinct when the model was applied to the mid-life cohort. In this case, the prediction of AD was enhanced by nearly 20% (NRI: 19.6% *p* = 0.008, IDI: 0.02% *p* = 0.02). Moreover, Cox regression analyses showed a significant association between PRS and AD. This stands to reason, given that Kunkles’ PRS was developed based on a cohort that included only AD cases [28]. Our findings indicate that Kunkles’ PRS is unsuitable for a general dementia risk assessment. Adding a PRS to the CAIDE model might only be helpful for specific risk prediction of AD in mid-life. This is also supported and might be explained by a recent report of young AD patients having fewer co-pathologies in addition to AD, which leads to a more accurate risk prediction [39]. Another reason might be a genetic difference which was assumed by Gunn and colleagues showing that a PRS for AD is not predictive of dementia in long-living individuals compared to controls [40].

Moreover, our results also indicate that Kunkles’ PRS does not relevantly improve the prediction over CAIDE Model 2, including *APOE* ε4. Thus, the predictive value of other SNPs, in addition to the *APOE* ε4 polymorphism in PRSs, is questionable. *APOE* is a well-known risk factor for dementia, especially AD, and a fundamental component in most dementia risk prediction models [5, 41, 42]. In our study, *APOE* ε4 carrier status was one of the strongest predictors. Interestingly, not only the additional predictive value of PRS but also of SCD seemed negligible when APOE was part of CAIDE Model 2. This again emphasises the strength of *APOE* in dementia risk prediction.

When applied to the mid-life and late-life subgroups, the CAIDE model showed a clearly higher discriminative ability for all three outcomes in the mid-life subgroup. This has also been reported in previous studies replicating or applying the CAIDE model [3, 43] and is in line with the CAIDE model initially developed in a mid-life cohort. More research is needed about risk factors that can improve dementia risk prediction in older individuals.

### Strengths and limitations

Strengths of this study include its prospective cohort design, a high number of participants, an extensive follow-up period of 17 years and its comparability to the German healthcare setting.

Nevertheless, limitations encompass a possibility of under- or misdiagnosis of dementia and dementia subtypes due to the community-based setting of the ESTHER study. In the ESTHER study, dementia diagnoses are made heterogeneously, and subtypes were often not diagnosed. Although this also reflects the reality of a community-based setting, which enhances the generalizability of the study and might explain the relatively low number of AD diagnoses, a possible under- or misdiagnosis of dementia might lead to an underestimation of results and may impact the strength and precision of prediction models created.

Given the recalibration of the CAIDE model to the ESTHER study, the direct comparability to the CAIDE model is affected. In addition, since our study population is mainly of European descent aged 50 to 75 at baseline, results cannot be applied to other ethnicities and age groups.

### Conclusion

This study showed that although AUCs only marginally increased when SCD and PRS were added to the CAIDE model, reclassification analyses reveal a statistically significant improvement in the model’s prediction accuracy. Adding SCD to the CAIDE model significantly improved the prediction of all-cause dementia and VD. In contrast, the addition of PRS statistically significantly improved the discriminative ability for AD, especially in mid-life. This represents an essential difference in terms of clinical translation. Since information on SCD can be more easily assessed than the calculation of PRS, this constitutes a major advantage. However, both a PRS and SCD seem to be of limited, if any, predictive value if information on *APOE* ε4 carrier status is available.

### Electronic supplementary material

Below is the link to the electronic supplementary material.

Supplementary Material 1

## Data Availability

The data that support the findings of this study are not openly available due to reasons of sensitivity and are available from the corresponding author upon reasonable request. Data are located in controlled access data storage at the German Cancer Research Center.

## Abbreviations

AD: Alzheimer’s disease
AIC: Akaike’s information criterion
APOE: Apolipo-protein
AUC: Area under the curve
CAIDE: Cardiovascular risk factors aging and dementia
CI: Confidence interval
ESTHER: Epidemiologische Studie zu Chancen der Verhütung, Früherkennung und optimierten Therapie chronischer Erkrankungen in der älteren Bevölkerung
GPs: General practitioners
GWAS: Genome-wide association studies
IDI: Integrated discrimination improvement
MCI: Mild cognitive impairment
MMSE: Mini-Mental State Examination
NRI: Net reclassification improvement
PRS: Polygenic risk score
ROC: Receiver operating characteristic
SAGE: Self-administered Gerocognitive Examination
SAS: Statistical Analysis System
SCD: Subjective cognitive decline
SD: Standard deviation
SNP: Single-nucleotide polymorphism
VD: Vascular dementia

## References

[1] Global status report. On the public health response to dementia. Word Health Organization; 2021.

[2] Livingston G, Huntley J, Liu KY, Costafreda SG, Selbæk G, Alladi S, et al. Dementia prevention, intervention, and care: 2024 report of the Lancet standing commission. The Lancet. 2024;404(10452):572–628. 39096926 10.1016/S0140-6736(24)01296-0

[3] Kivimäki M, Livingston G, Singh-Manoux A, Mars N, Lindbohm JV, Pentti J, et al. Estimating dementia risk using multifactorial prediction models. JAMA Netw Open. 2023;6(6):e2318132–e. 37310738 10.1001/jamanetworkopen.2023.18132 PMC10265307

[4] Licher S, Yilmaz P, Leening MJG, Wolters FJ, Vernooij MW, Stephan BCM, et al. External validation of four dementia prediction models for use in the general community-dwelling population: a comparative analysis from the Rotterdam Study. Eur J Epidemiol. 2018;33(7):645–55. 29740780 10.1007/s10654-018-0403-y PMC6061119

[5] Hou X-H, Feng L, Zhang C, Cao X-P, Tan L, Yu J-T. Models for predicting risk of dementia: a systematic review. J Neurol Neurosurg Psychiatry. 2019;90(4):373–9. 29954871 10.1136/jnnp-2018-318212

[6] John LH, Kors JA, Fridgeirsson EA, Reps JM, Rijnbeek PR. External validation of existing dementia prediction models on observational health data. BMC Med Res Methodol. 2022;22(1):311. 36471238 10.1186/s12874-022-01793-5 PMC9720950

[7] Goerdten J, Čukić I, Danso SO, Carrière I, Muniz-Terrera G. Statistical methods for dementia risk prediction and recommendations for future work: a systematic review. Alzheimer’s Dementia: Translational Res Clin Interventions. 2019;5(1):563–9. 10.1016/j.trci.2019.08.001 PMC6804431 31646170

[8] Anstey KJ, Zheng L, Peters R, Kootar S, Barbera M, Stephen R et al. Dementia risk scores and their role in the implementation of risk reduction guidelines. Front Neurol. 2022;12. 10.3389/fneur.2021.765454 PMC8764151 35058873

[9] Kivipelto M, Ngandu T, Laatikainen T, Winblad B, Soininen H, Tuomilehto J. Risk score for the prediction of dementia risk in 20 years among middle aged people: a longitudinal, population-based study. Lancet Neurol. 2006;5(9):735–41. 16914401 10.1016/S1474-4422(06)70537-3

[10] Möllers T, Stocker H, Perna L, Rujescu D, Holleczek B, Schöttker B et al. Subjective short-term memory difficulties at ages 50–75 predict dementia risk in a community-based cohort followed over 17 years. Age Ageing. 2022;51(6). 10.1093/ageing/afac113 35697354

[11] Jessen F, Amariglio RE, Buckley RF, van der Flier WM, Han Y, Molinuevo JL, et al. The characterisation of subjective cognitive decline. Lancet Neurol. 2020;19(3):271–8. 31958406 10.1016/S1474-4422(19)30368-0 PMC7062546

[12] Robertson FE, Jacova C. A systematic review of subjective cognitive characteristics predictive of longitudinal outcomes in older adults. Gerontologist. 2022. 10.1093/geront/gnac109 35908232

[13] Rabin LA, Smart CM, Amariglio RE. Subjective cognitive decline in preclinical Alzheimer’s Disease. Annu Rev Clin Psychol. 2017;13:369–96. 28482688 10.1146/annurev-clinpsy-032816-045136

[14] Clark K, Leung YY, Lee WP, Voight B, Wang LS. Polygenic risk scores in Alzheimer’s Disease Genetics: methodology, applications, inclusion, and Diversity. J Alzheimers Dis. 2022;89(1):1–12. 35848019 10.3233/JAD-220025 PMC9484091

[15] Leonenko G, Baker E, Stevenson-Hoare J, Sierksma A, Fiers M, Williams J, et al. Identifying individuals with high risk of Alzheimer’s disease using polygenic risk scores. Nat Commun. 2021;12(1):4506. 34301930 10.1038/s41467-021-24082-z PMC8302739

[16] Baker E, Escott-Price V. Polygenic Risk Scores in Alzheimer’s Disease: Current Applications and Future Directions. Front Digit Health. 2020;2. 10.3389/fdgth.2020.00014 PMC8521998 34713027

[17] Stocker H, Perna L, Weigl K, Möllers T, Schöttker B, Thomsen H, et al. Prediction of clinical diagnosis of Alzheimer’s disease, vascular, mixed, and all-cause dementia by a polygenic risk score and APOE status in a community-based cohort prospectively followed over 17 years. Mol Psychiatry. 2021;26(10):5812–22. 32404947 10.1038/s41380-020-0764-y PMC8758470

[18] Escott-Price V, Sims R, Bannister C, Harold D, Vronskaya M, Majounie E, et al. Common polygenic variation enhances risk prediction for Alzheimer’s disease. Brain. 2015;138(12):3673–84. 26490334 10.1093/brain/awv268 PMC5006219

[19] Chaudhury S, Patel T, Barber IS, Guetta-Baranes T, Brookes KJ, Chappell S, et al. Polygenic risk score in postmortem diagnosed sporadic early-onset Alzheimer’s disease. Neurobiol Aging. 2018;62:244e1. e1-.e8. 10.1016/j.neurobiolaging.2017.09.035 PMC5995122 29103623

[20] Trares K, Bhardwaj M, Perna L, Stocker H, Petrera A, Hauck SM, et al. Association of the inflammation-related proteome with dementia development at older age: results from a large, prospective, population-based cohort study. Alzheimers Res Ther. 2022;14(1):128. 36085081 10.1186/s13195-022-01063-y PMC9461133

[21] Stocker H, Beyer L, Trares K, Perna L, Rujescu D, Holleczek B, et al. Association of kidney function with development of Alzheimer Disease and other dementias and dementia-related blood biomarkers. JAMA Netw Open. 2023;6(1):e2252387. 36692879 10.1001/jamanetworkopen.2022.52387 PMC10408272

[22] Löw M, Stegmaier C, Ziegler H, Rothenbacher D, Brenner H. [Epidemiological investigations of the chances of preventing, recognizing early and optimally treating chronic diseases in an elderly population (ESTHER study)]. Dtsch Med Wochenschr. 2004;129(49):2643–7. 15578318 10.1055/s-2004-836089

[23] Royston P, Sauerbrei W. Building multivariable regression models with continuous covariates in clinical epidemiology–with an emphasis on fractional polynomials. Methods Inf Med. 2005;44(4):561–71. 16342923 10.1055/s-0038-1634008

[24] Anderson CA, Pettersson FH, Clarke GM, Cardon LR, Morris AP, Zondervan KT. Data quality control in genetic case-control association studies. Nat Protoc. 2010;5(9):1564–73. 21085122 10.1038/nprot.2010.116 PMC3025522

[25] Das S, Forer L, Schönherr S, Sidore C, Locke AE, Kwong A, et al. Next-generation genotype imputation service and methods. Nat Genet. 2016;48(10):1284–7. 27571263 10.1038/ng.3656 PMC5157836

[26] McCarthy S, Das S, Kretzschmar W, Delaneau O, Wood AR, Teumer A, et al. A reference panel of 64,976 haplotypes for genotype imputation. Nat Genet. 2016;48(10):1279–83. 27548312 10.1038/ng.3643 PMC5388176

[27] Stocker H, Perna L, Weigl K, Möllers T, Schöttker B, Thomsen H et al. Prediction of clinical diagnosis of Alzheimer’s disease, vascular, mixed, and all-cause dementia by a polygenic risk score and APOE status in a community-based cohort prospectively followed over 17 years. Mol Psychiatry. 2020. 10.1038/s41380-021-01311-x PMC8758469 34599279

[28] Kunkle BW, Grenier-Boley B, Sims R, Bis JC, Damotte V, Naj AC, et al. Genetic meta-analysis of diagnosed Alzheimer’s disease identifies new risk loci and implicates Aβ, tau, immunity and lipid processing. Nat Genet. 2019;51(3):414–30. 30820047 10.1038/s41588-019-0358-2 PMC6463297

[29] May S, Hosmer DW. A cautionary note on the use of the Grønnesby and Borgan goodness-of-fit test for the Cox proportional hazards model. Lifetime Data Anal. 2004;10(3):283–91. 15456108 10.1023/B:LIDA.0000036393.29224.1d

[30] Pencina MJ, D’Agostino RB, Sr., D’Agostino RB Jr., Vasan RS. Evaluating the added predictive ability of a new marker: from area under the ROC curve to reclassification and beyond. Stat Med. 2008;27(2):157–72. discussion 207 – 12. 17569110 10.1002/sim.2929

[31] Pencina MJ, D’Agostino RB, Sr., Demler OV. Novel metrics for evaluating improvement in discrimination: net reclassification and integrated discrimination improvement for normal variables and nested models. Stat Med. 2012;31(2):101–13. 22147389 10.1002/sim.4348 PMC3341978

[32] Scharre DW, Chang S-I, Murden RA, Lamb J, Beversdorf DQ, Kataki M, et al. Self-administered gerocognitive examination (SAGE): a brief cognitive Assessment instrument for mild cognitive impairment (MCI) and early dementia. Alzheimer Disease Assoc Disorders. 2010;24(1):64–71. 10.1097/WAD.0b013e3181b03277 20220323

[33] Scharre DW, Chang SI, Nagaraja HN, Wheeler NC, Kataki M. Self-administered gerocognitive examination: longitudinal cohort testing for the early detection of dementia conversion. Alzheimers Res Ther. 2021;13(1):192. 34872596 10.1186/s13195-021-00930-4 PMC8650250

[34] Silvan Licher MD, Maarten JG, Leening MD,, PD, Pinar Yilmaz MD, Wolters FJ,, MDPD, Heeringa J,, MDPD, Patrick JE, Bindels MD,, PD, et al. Development and validation of a dementia risk prediction model in the General Population: an analysis of three longitudinal studies. Am J Psychiatry. 2019;176(7):543–51. 10.1176/appi.ajp.2018.18050566 30525906

[35] Verhaaren BFJ, Vernooij MW, Koudstaal PJ, Uitterlinden AG, van Duijn CM, Hofman A, et al. Alzheimer’s Disease genes and cognition in the Nondemented General Population. Biol Psychiatry. 2013;73(5):429–34. 22592056 10.1016/j.biopsych.2012.04.009

[36] Earl Robertson F, Jacova C. A systematic review of subjective cognitive characteristics predictive of longitudinal outcomes in older adults. Gerontologist. 2023;63(4):700–16. 35908232 10.1093/geront/gnac109

[37] Pike KE, Cavuoto MG, Li L, Wright BJ, Kinsella GJ. Subjective cognitive decline: level of risk for future dementia and mild cognitive impairment, a Meta-Analysis of Longitudinal studies. Neuropsychol Rev. 2022;32(4):703–35. 34748154 10.1007/s11065-021-09522-3

[38] Jessen F, Amariglio RE, van Boxtel M, Breteler M, Ceccaldi M, Chételat G, et al. A conceptual framework for research on subjective cognitive decline in preclinical Alzheimer’s disease. Alzheimer’s Dement J Alzheimer’s Assoc. 2014;10(6):844–52. 10.1016/j.jalz.2014.01.001 PMC4317324 24798886

[39] Robinson JL, Xie SX, Baer DR, Suh E, Van Deerlin VM, Loh NJ, et al. Pathological combinations in neurodegenerative disease are heterogeneous and disease-associated. Brain. 2023;146(6):2557–69. 36864661 10.1093/brain/awad059 PMC10232273

[40] Gunn S, Wainberg M, Song Z, Andersen S, Boudreau R, Feitosa MF, et al. Distribution of 54 polygenic risk scores for common diseases in long lived individuals and their offspring. GeroScience. 2022;44(2):719–29. 35119614 10.1007/s11357-022-00518-2 PMC9135909

[41] Tang EYH, Harrison SL, Errington L, Gordon MF, Visser PJ, Novak G, et al. Current developments in Dementia Risk Prediction Modelling: an updated systematic review. PLoS ONE. 2015;10(9):e0136181. 26334524 10.1371/journal.pone.0136181 PMC4559315

[42] Hall A, Pekkala T, Polvikoski T, van Gils M, Kivipelto M, Lötjönen J, et al. Prediction models for dementia and neuropathology in the oldest old: the Vantaa 85 + cohort study. Alzheimers Res Ther. 2019;11(1):11. 30670070 10.1186/s13195-018-0450-3 PMC6343349

[43] Anstey KJ, Cherbuin N, Herath PM, Qiu C, Kuller LH, Lopez OL, et al. A self-report risk index to predict occurrence of Dementia in three independent cohorts of older adults: the ANU-ADRI. PLoS ONE. 2014;9(1):e86141. 24465922 10.1371/journal.pone.0086141 PMC3900468

