# Supplementary material for: Comparison of subjective cognitive decline and polygenic risk score in the prediction of all-cause dementia, Alzheimer’s disease and vascular dementia
Source: Alzheimers Res Ther. 2024 Aug 19;16:188. doi: 10.1186/s13195-024-01559-9 (PMC11331600; doi:10.1186/s13195-024-01559-9)
Supplement: Supplementary file 1 — Supplementary Material 1 [file 13195_2024_1559_MOESM1_ESM.docx]

**Supplemental Material to Article**

“**Comparison of subjective cognitive decline and polygenic risk score in the prediction of all-cause dementia, Alzheimer’s disease and vascular dementia**”

Contents

[Supplemental Table 1. Baseline characteristics of included (n=5,360) and excluded (n=4,580) study participants. 2](#_Toc112748049)

[Supplemental Table 2. Associations of CAIDE model variables with all-cause dementia. 3](#_Toc112748050)

[Supplemental Table 3. Associations of CAIDE model variables with Alzheimer’s disease. 4](#_Toc112748051)

[Supplemental Table 4. Associations of CAIDE model variables with vascular dementia. 5](#_Toc112748052)

# Supplemental Table 1. Baseline characteristics of included (n=5,360) and excluded (n=4,580) study participants.

| **Baseline characteristics** | **Included study participants  (n=5,360)** | **Excluded study participants (n=4,580)** |
| --- | --- | --- |
| **Age (years), mean (SD)** | 61.62 (6.5) | 62.73 (6.7) |
| Mid-life (50-64 years), n (%) | 3458 (64.5) | 2646 (57.8) |
| Late-life (65-75 years), n (%) | 1902 (35.5) | 1934 (42.2) |
| **Education (years), n(%)** |  |  |
| <9 | 3923 (73.2) | 3312 (76.5) |
| 9-11 | 790 (14.7) | 582 (13.5) |
| ≥12 | 647 (12.1) | 434 (10.0) |
| **Sex, n (%)** |  |  |
| Female | 2883 (53.8) | 2579 (56.3) |
| Male | 2477 (46.2) | 2001 (43.7) |
| **SBP (mmHg), mean (SD)** | 139.19 (19.5) | 140.89 (19.6) |
| **BMI (kg/m^2^), mean (SD)** | 27.66 (4.3) | 27.71 (4.4) |
| **Total cholesterol (mmol/L),  mean (SD)** | 5.69 (1.3) | 5.66 (1.3) |
| **Physical activity ^a^, n (%)** |  |  |
| Inactive | 938 (17.5) | 1181 (26.0) |
| Low | 2468 (46.0) | 2058 (45.2) |
| Medium or high | 1954 (36.5) | 1311 (28.8) |
| ***APOE* genotypes, n (%)** |  |  |
| ε4 non-carrier | 3953 (73.8) | 3000 (74.2) |
| ε4 carrier | 1407 (26.2) | 1045 (25.8) |

Abbreviations: CI, confidence interval; BMI, body mass index; *APOE*

^a^“Inactive” was defined by < 1 h of vigorous or < 1 h light physical activity per week. “Medium or high” was defined by ≥ 2 h of vigorous and ≥ 2 h of light physical activity/week. All other amounts of physical activity were grouped into the category “Low”.

Supplemental Table 2. Associations of CAIDE model variables with all-cause dementia.

| **CAIDE model variables** | **CAIDE model 1**^a^ | |  | **CAIDE model 2**^b^ | |
| --- | --- | --- | --- | --- | --- |
|  | **Hazard Ratio (95%CI)^a^** | **p-value** |  | **Hazard Ratio (95%CI)^b^** | **p-value** |
| **Age (years)** | **1.18 (1.16-1.21)** | **<0.001** |  | **1.18 (1.16-1.21)** | **<0.001** |
| **Education (years)** |  |  |  |  |  |
| <9 | 1.00 Ref. |  |  | 1.00 Ref. |  |
| 9-11 | **0.60 (0.43-0.84)** | **0.003** |  | **0.62 (0.44-0.87)** | **0.005** |
| ≥12 | **0.72 (0.51-1.00)** | **0.049** |  | **0.71 (0.51-0.99)** | **0.045** |
| **Sex** |  |  |  |  |  |
| Female | 1.00 Ref. |  |  | 1.00 Ref. |  |
| Male | **1.40 (1.15-1.72)** | **0.001** |  | **1.39 (1.13-1.70)** | **0.002** |
| **SBP (mmHg)** | 1.00 (1.00-1.01) | 0.456 |  | 1.00 (1.00-1.01) | 0.472 |
| **BMI (kg/m^2^)** | 0.99 (0.96-1.01) | 0.251 |  | 0.99 (0.96-1.02) | 0.452 |
| **Total cholesterol (mmol/L)** | 0.98 (0.91-1.06) | 0.678 |  | 0.97 (0.91-1.05) | 0.476 |
| **Physical activity ^c^** |  |  |  |  |  |
| Inactive | 1.00 Ref. |  |  | 1.00 Ref. |  |
| Low | **0.72 (0.57-0.92)** | **0.008** |  | **0.73 (0.57-0.93)** | **0.010** |
| Medium or high | **0.62 (0.47-0.82)** | **0.001** |  | **0.63 (0.48-0.83)** | **0.001** |
| ***APOE* genotypes** |  |  |  |  |  |
| ε4 non-carrier | **-** | **-** |  | 1.00 Ref. |  |
| ε4 carrier | **-** | **-** |  | **2.07 (1.70-2.52)** | **<0.001** |

Note: Numbers printed in bold are statistically significant.

Abbreviations: CI, Confidence Interval; *APOE*, apolipoprotein E.

^a^The CAIDE model 1 includes age, education, sex, systolic blood pressure, body-mass index, total cholesterol and physical activity.

^b^The CAIDE model 2 includes the variables of CAIDE model 1 and *APOE* ε4 status.

^c^“Inactive” was defined by < 1 h of vigorous or < 1 h light physical activity per week. “Medium or high” was defined by ≥ 2 h of vigorous and ≥ 2 h of light physical activity/week. All other amounts of physical activity were grouped into the category “Low”.

Supplemental Table 3. Associations of CAIDE model variables with Alzheimer’s disease.

| **CAIDE model variables** | **CAIDE model 1^a^** | |  | **CAIDE model 2^b^** | |
| --- | --- | --- | --- | --- | --- |
|  | **Hazard Ratio (95%CI)^a^** | **p-value** |  | **Hazard Ratio (95%CI)^b^** | **p-value** |
| **Age (years)** | **1.18 (1.14-1.22)** | **<0.001** |  | **1.18 (1.14-1.22)** | **<0.001** |
| **Education (years)** |  |  |  |  |  |
| <9 | 1.00 Ref. |  |  | 1.00 Ref. |  |
| 9-11 | 0.59 (0.33-1.05) | 0.075 |  | 0.62 (0.35-1.11) | 0.109 |
| ≥12 | 0.59 (0.31-1.10) | 0.098 |  | 0.58 (0.31-1.10) | 0.097 |
| **Sex** |  |  |  |  |  |
| Female | 1.00 Ref. |  |  | 1.00 Ref. |  |
| Male | 1.23 (0.86-1.74) | 0.256 |  | 1.20 (0.84-1.70) | 0.321 |
| **SBP (mmHg)** | 1.00 (0.99-1.01) | 0.628 |  | 1.00 (0.99-1.01) | 0.607 |
| **BMI (kg/m^2^)** | 0.95 (0.91-1.00) | 0.037 |  | 0.96 (0.92-1.01) | 0.079 |
| **Total cholesterol (mmol/L)** | 0.99 (0.87-1.12) | 0.822 |  | 0.97 (0.86-1.10) | 0.649 |
| **Physical activity ^c^** |  |  |  |  |  |
| Inactive | 1.00 Ref. |  |  | 1.00 Ref. |  |
| Low | **0.57 (0.38-0.85)** | **0.006** |  | **0.57 (0.38-0.85)** | **0.006** |
| Medium or high | **0.53 (0.34-0.84)** | **0.007** |  | **0.54 (0.34-0.86)** | **0.009** |
| ***APOE* genotypes** |  |  |  |  |  |
| ε4 non-carrier | - | - |  | 1.00 Ref. |  |
| ε4 carrier | - | - |  | **3.08 (2.21-4.30)** | **<0.001** |

Note: Numbers printed in bold are statistically significant.

Abbreviations: CI, Confidence Interval; APOE, apolipoprotein E; SBP, systolic blood pressure; BMI, body mass index.

^a^The CAIDE model 1 includes age, education, sex, systolic blood pressure, body-mass index, total cholesterol and physical activity.

^b^The CAIDE model 2 includes the variables of CAIDE model 1 and *APOE* ε4 status.

^c^“Inactive” was defined by < 1 h of vigorous or < 1 h light physical activity per week. “Medium or high” was defined by ≥ 2 h of vigorous and ≥ 2 h of light physical activity/week. All other amounts of physical activity were grouped into the category “Low”.

Supplemental Table 4. Associations of CAIDE model variables with vascular dementia.

| **CAIDE model variables** | **CAIDE model 1^a^** | |  | **CAIDE model 2^b^** | |
| --- | --- | --- | --- | --- | --- |
|  | **Hazard Ratio (95%CI)^a^** | **p-value** |  | **Hazard Ratio (95%CI)^b^** | **p-value** |
| **Age (years)** | **1.19 (1.16-1.23)** | **<0.0001** |  | **1.19 (1.16-1.23)** | **<0.001** |
| **Education (years)** |  |  |  |  |  |
| <9 | 1.00 Ref. |  |  | 1.00 Ref. |  |
| 9-11 | 0.66 (0.39-1.12) | 0.121 |  | 0.67 (0.40-1.14) | 0.110 |
| ≥12 | 0.62 (0.35-1.09) | 0.097 |  | 0.61 (0.35-1.08) | 0.092 |
| **Sex** |  |  |  |  |  |
| Female | 1.00 Ref. |  |  | 1.00 Ref. |  |
| Male | **1.54 (1.10-2.15)** | **0.011** |  | **1.53 (1.10-2.14)** | **0.013** |
| **SBP (mmHg)** | 1.00 (1.00-1.01) | 0.483 |  | 1.00 (0.99-1.01) | 0.509 |
| **BMI (kg/m^2^)** | 0.99 (0.95-1.04) | 0.744 |  | 1.00 (0.95-1.04) | 0.869 |
| **Total cholesterol (mmol/L)** | 0.98 (0.87-1.11) | 0.756 |  | 0.98 (0.86-1.10) | 0.695 |
| **Physical activity ^c^** |  |  |  |  |  |
| Inactive | 1.00 Ref. |  |  | 1.00 Ref. |  |
| Low | 0.87 (0.57-1.32) | 0.519 |  | 0.88 (0.58-1.33) | 0.531 |
| Medium or high | 0.77 (0.48-1.22) | 0.257 |  | 0.77 (0.48-1.22) | 0.265 |
| ***APOE* genotypes** |  |  |  |  |  |
| ε4 non-carrier | - | - |  | 1.00 Ref. |  |
| ε4 carrier | - | - |  | **1.67 (1.20-2.33)** | **0.003** |

Note: Numbers printed in bold are statistically significant.

Abbreviations: CI, Confidence Interval; APOE, apolipoprotein E; SBP, systolic blood pressure; BMI, body mass index.

^a^The CAIDE model 1 includes age, education, sex, systolic blood pressure, body-mass index, total cholesterol and physical activity.

^b^The CAIDE model 2 includes the variables of CAIDE model 1 and *APOE* ε4 status.

^c^“Inactive” was defined by < 1 h of vigorous or < 1 h light physical activity per week. “Medium or high” was defined by ≥ 2 h of vigorous and ≥ 2 h of light physical activity/week. All other amounts of physical activity were grouped into the category “Low”.
